# Diagnostic and Severity Assessment of Coronary Artery Disease Using ApoB/ApoA-I Ratio: Insights from a Statin-Treated Eastern European Cohort

**DOI:** 10.3390/medicina62020297

**Published:** 2026-02-02

**Authors:** Raul-Alexandru Jigoranu, Ovidiu Mitu, Alexandru Florinel Oancea, Radu-Stefan Miftode, Ana Maria Buburuz, Amin Bazyani, Radu-Sebastian Gavril, Theodor-Constantin Stamate, Cristina Andreea Adam, Ionela-Larisa Miftode, Antoniu Octavian Petris, Irina-Iuliana Costache Enache, Florin Mitu

**Affiliations:** 1 Grigore T. Popa University of Medicine and Pharmacy Iasi, 700115 Iasi, Romania; alexandru.jigoranu@umfiasi.ro (R.-A.J.); ovidiu.mitu@umfiasi.ro (O.M.); alexandru.oancea@umfiasi.ro (A.F.O.); ana-maria.buburuz@umfiasi.ro (A.M.B.); aminbazyani@gmail.com (A.B.); rgavril87@yahoo.com (R.-S.G.); stamate.theodor@gmail.com (T.-C.S.); adam.cristina93@gmail.com (C.A.A.); ionela-larisa.miftode@umfiasi.ro (I.-L.M.); antoniu.petris@umfiasi.ro (A.O.P.); irina.costache@umfiasi.ro (I.-I.C.E.); mitu.florin@yahoo.com (F.M.); 2 Department of Cardiology, “St. Spiridon” Emergency County Hospital, 700111 Iasi, Romania; 3 Department of Cardiovascular Rehabilitation, Clinical Rehabilitation Hospital, 700661 Iasi, Romania

**Keywords:** apolipoprotein B, apolipoprotein A-I, apoB/apoA ratio, Lp(a), chronic coronary syndrome, atherosclerosis, biomarkers

## Abstract

*Background and Objectives*: Atherosclerosis continues to be a major determinant of the global health burden, with ischemic heart disease representing one of the leading causes of morbidity and mortality worldwide. Although cardiovascular (CV) prevention strategies focus on pro-atherogenic lipoproteins, such as LDL-C, non-HDL-C, and apoB, the balance between atherogenic and anti-atherogenic lipoproteins may better reflect the overall atherogenic burden. Apolipoprotein B (apoB) reflects the total number of circulating atherogenic particles, whereas apolipoprotein A-I (apoA) is the main protein component of HDL, the major anti-atherogenic lipoprotein. Integrating these two parameters into the apoB/apoA ratio results in a composite biomarker that reflects this balance. In this study, we aimed to evaluate whether the apoB/apoA ratio can predict the presence and the severity of coronary artery disease (CAD) in a cohort from an Eastern European hospital, under moderate-intensity statin treatment. Additionally, we assessed whether lipoprotein(a) [Lp(a)] provides any additional diagnostic value. *Materials and Methods*: We consecutively enrolled 121 statin-treated patients, who presented for elective invasive coronary angiography. Patients with history of coronary revascularization or acute coronary syndrome were excluded. The study cohort was further divided into two groups, according to the severity of coronary stenosis: 69 patients with non-significant CAD (N-CAD) and 52 patients with hemodynamically significant CAD (S-CAD). Apolipoprotein B, apolipoprotein A-I, and lipoprotein(a) were measured using a standardized immunoturbidimetric assay, at the moment of enrollment. The severity of coronary stenosis was measured using Quantitative Coronary Analysis (QCA) software and the total coronary atherosclerotic burden of each patient was quantified using the Gensini score. *Results*: The apoB/apoA ratio was significantly higher in the S-CAD groups, compared with N-CAD patients (0.53 ± 0.16 vs. 0.73 ± 0.18). Furthermore, in the apoB/apoA-based analysis, the Gensini score increased progressively across the three tertiles (8.55 ± 19.60 vs. 14.57 ± 21.65 vs. 29.8 ± 27.78, *p* = 0.000) and so did the percentage of patients with three-vessel disease (5% vs. 19.5% vs. 32.5%, *p* = 0.000) and left main disease (5% vs. 7.3% vs. 20%, *p* = 0.031). The apoB/apoA ratio showed a significant correlation with the severity of CAD, as expressed by the Gensini score (r = 0.513, *p* < 0.001, 95% CI: 0.357–0.641). The association between apoB/apoA ratio and the presence and severity of CAD expanded beyond group comparison. In the logistic regression, this biomarker proved to be a valuable predictor for S-CAD (per SD increase: OR 2.509, 95% CI: 1.441–4.369, *p* = 0.001), three-vessel disease (per SD increase: OR 2.339, 95% CI: 1.427–3.892, *p* = 0.001), and left main disease (per SD increase: OR 2.771, 95% CI: 1.489–5.156, *p* = 0.001). The apoB/apoA ratio remained significant after adjusting for other CV risk factors and independent to LDL-C, as shown by the analysis that we performed among the lowest LDL-C tertile patients. Participants with S-CAD showed higher concentrations of Lp(a). However, adding this lipoprotein to the multivariate analysis, resulted only in a marginal improvement in the predictive power. *Conclusions*: The ApoB/apoA ratio emerged as an independent predictor for hemodynamically significant coronary stenosis and for CAD severity. Additionally, higher apoB/apoA values were associated with anatomical high-risk features, such as three-vessel disease or left main disease. In contrast, Lp(a) did not provide a substantial increase in the predictive power of multivariate models in this stable CAD cohort.

## 1. Introduction

Atherosclerosis is a complex inflammatory disease, responsible for a wide range of pathologies, including coronary artery disease (CAD), stroke, and peripheral artery disease (PAD). Despite recent advances in primary and secondary prevention protocols, atherosclerosis continues to be the main determinant of global health burden as, except for in the COVID-19 era, ischemic heart disease and stroke are the leading two causes of morbidity and mortality worldwide [1].

In the physiopathological process of atherosclerosis, there are three main determinants that form a strong and interdependent triad: dyslipidemia, endothelial dysfunction, and inflammation [2]. Each component of this triad is indispensable. Endothelial dysfunction and inflammation form the foundation, as they are essential for initiating and amplifying atherosclerosis, whereas lipid particles represent the core constituents of the atherosclerotic plaques, exerting a quantitatively linear relationship with this process [3]. This is the reason why cardiovascular (CV) prevention protocols worldwide are mainly focused on lowering serum atherogenic particles. Traditionally, lipid lowering therapies target conventional lipid biomarkers like low-density lipoprotein cholesterol (LDL-C) and triglycerides (TG). Despite achieving the target values for LDL-C and TG, some patients continue to experience fatal or non-fatal CV events, which led to the concept of residual CV risk. Hence, in 2019, The European Society of Cardiology (ESC) introduced non-high-density lipoprotein cholesterol (non-HDL-C) and apolipoprotein B (apoB) as secondary treatment targets [4]. Even though LDL-C remains the main lipidic CV risk assessment tool in the latest updated ESC guidelines [5], increasing evidence support the superiority of non-HDL-C and even the supremacy of apoB [6]. ApoB is an amphipathic protein, synthetized by the liver, that constitutes the core framework for all atherogenic particles [2]. It has an essential role in atherosclerosis, as its positively charged amino acid domains facilitate lipoprotein retention in the subendothelial space by binding to the negatively charged proteoglycans. Considering that each lipoprotein contains a single apoB molecule, measuring serum apoB gives a clear view over the total number of circulating atherogenic particles [7]. Over the years, a plethora of studies linked apoB levels to CV risk [8,9], coronary atherosclerosis [10], and PAD [11], all of which transformed this biomarker into a valuable contender for LDL-C.

While current guidelines mostly emphasize the risk inflicted by atherogenic lipoproteins, considerably less attention has been given to the protective role of anti-atherogenic particles. High-density lipoprotein (HDL) is the main anti-atherogenic lipoprotein, which exerts an antagonistic effect, as compared to other lipoproteins implicated in the atherosclerosis. Contrary to LDL, HDL is known for its “reverse cholesterol transport” properties, as it has been demonstrated to extract excess cholesterol from peripheral vasculature and remove it from the body via hepatobiliary tract [12]. Several epidemiological studies linked low levels of HDL cholesterol (HDL-C) to an increased CV risk; however, very high levels proved to have a similar effect, suggesting that there is a U-shaped relationship between HDL-C and CV mortality [13]. HDL composition is highly complex, comprising over 14 types of apolipoproteins, essential for its structural integrity and functions. Among these, apolipoprotein A-1 (apoA) accounts for approximately 70% of the total protein content, being the most relevant apolipoprotein from HDL composition [14]. Even though current guidelines do not consider the protective effect of apoA, several epidemiological and case-control studies support its utility for CV risk assessment [15,16] and even its superiority over HDL-C [17]; however, the available data is still conflicting [18].

Secondary prevention studies showed that low levels of anti-atherogenic lipoproteins are predictive for recurrent CV events, despite optimal LDL-C levels [19], underlying the importance of the balance between atherogenic and anti-atherogenic particles. The apoB/apoA ratio is increasingly recognized as a solid marker reflecting this balance, as it integrates the total number of atherogenic particles (the apoB component) and anti-atherogenic lipoproteins (apoA component). Large-scale studies, including INTERHEART and AMORIS cohorts, demonstrated that an elevated apoB/apoA ratio is more predictive of myocardial infarction and CV mortality, when compared to traditional lipid biomarkers and other CV risk factors [20,21]. Moreover, intravascular imaging studies linked elevated apoB/apoA ratio to atherosclerotic plaque vulnerability, further supporting the findings from case-control studies [22]. Lastly, in acute coronary syndrome patients, apoB/apoA ratio correlated with the severity of CAD, supporting the quantitative relationship with plaque burden [23]. All these findings highlight the strong association between apoB/apoA ratio, CV risk, and CAD, advocating its use as a clinically meaningful and potentially superior biomarker to traditional lipid parameters.

It is estimated that up to 70% of ischemic heart disease susceptibility is attributable to hereditary factors [24]. Beyond conventional lipid parameters and apolipoproteins, lipoprotein(a) (Lp(a)) emerged as an independent and genetically determined risk factor for CV disease. Structurally, Lp(a) consists of single molecules of a plasminogen-like glycoprotein, named apolipoprotein(a) (apo(a)), connected to a particle of LDL via disulfide bonds [25]. The size of apo(a) is the strongest determinant of Lp(a) production, with an inverse relationship observed between apo(a) isoform size and circulating Lp(a) concentration. Genetic variations in LPA gene, which encodes apo(a), account for approximately 90% of Lp(a) production rate, making this lipoprotein one of the major genetically determined CV risk factors [26]. Large-scale studies showed that increased Lp(a) levels are associated with a higher risk of major CV events (MACE), independent of LDL-C levels [27,28,29], creating a solid base for further studies that investigated the role of this lipoprotein as a CV risk indicator. The link between Lp(a) and risk of MACE arises not only from its pro-atherogenic properties [30], but also from its pro-thrombogenic potential. As concluded by intravascular imaging studies, higher Lp(a) levels are associated with vulnerable plaque features [31]. Additionally, after an acute coronary syndrome, extended dual antiplatelet therapy (DAPT) lowers the rates of stent thrombosis in patients with increased Lp(a) levels, supporting the link between this lipoprotein and the thrombotic risk [32].

In our study, we aimed to evaluate whether the apoB/apoA ratio can predict the presence and the severity of CAD in a statin-treated cohort from Eastern Europe. We also investigated whether Lp(a) provides additional diagnostic value in case of stable CAD.

## 2. Materials and Methods

### 2.1. Study Design, Patient Enrollment, and Investigations

We conducted a prospective case-control study, which included patients who presented electively between January 2024 and January 2025 to our hospital, for invasive coronary angiography. We only included patients over the age of 18, who were treated with moderate-intensity statins (Atorvastatin 10/20 mg or Rosuvastatin 10 mg) and who accepted the informed consent. As stated in our previously published study, which provided a comparative analysis between apoB and standard lipid biomarkers [33], patients with positive history of acute coronary syndrome (ACS), coronary revascularization, chronic inflammatory disease (excluding atherosclerosis), active neoplasia, chronic kidney disease (eGFR < 30 mL/min/1.73 m^2^), under treatment with other lipid-lowering therapies, except for statins, or liver cirrhosis, were excluded from the study. A total of 121 patients were enrolled and further divided into 2 groups, according to the CAD status: 52 patients with S-CAD (significant CAD, defined as stenosis ≥ 50% for left main or proximal anterior descending artery, or stenosis ≥ 70% for other coronary segments) and 69 patients with N-CAD (no coronary artery disease or lesions which did not meet the S-CAD criteria).

For each enrolled patient, we gathered both clinical and paraclinical information. Based on the clinical exam and anamnesis, we provided for all patients, information regarding their CV risk factors and most relevant comorbidities [body-mass index (BMI), smoking status, blood pressure, atrial fibrillation (AF), diabetes mellitus (DM), chronic kidney disease (CKD), chronic pulmonary obstructive disease (COPD), transient ischemic attack (TIA) and stroke]. The echocardiographic examination was performed using a GE Vivid V7 (General Electric, Boston, MA, USA) device. Epicardial fat thickness was measured in parasternal long-axis and parasternal short-axis view, over the right ventricular free wall. All determinations were performed at end-systole and the cut-off for pathological epicardial fat was considered ≥5 mm. The invasive coronary angiography was performed by experienced operators, using Azurion 7 Philips Image Guided Therapy System (Philips Healthcare, Best, The Netherlands). In selected cases, Fraction Flow Reserve (FFR) was performed in order to determine the hemodynamical significance of borderline coronary stenosis. Coronary stenosis severity was then measured by experienced interventional cardiologists using Quantitative Coronary Analysis (QCA) software (2D Quantitative Analysis, Philips Azurion platform Release 2.2, Philips Medical Systems, Best, The Netherlands) and according to the standard protocol. The total burden of coronary atherosclerosis for each patient was assessed using the Gensini score, according to the originally published method [34]. Each coronary lesion was graded according to the severity of stenosis (1 point for 1–25% stenosis, 2 points for 26–50% stenosis, 4 points for 51–75% stenosis, 8 points for 76–90% stenosis, 16 points for 91–99% stenosis, and 32 points for total occlusion) and then multiplied with a correction factor, specific to the diseased coronary segment (5 for the left main, 2.5 for the proximal anterior descending artery and proximal circumflex artery, 1.5 for mid anterior descending artery, 1 for distal anterior descending artery, right coronary artery, postero-lateral branches, and obtuse marginal branches, and 0.6 for other smaller branches).

For biochemical analysis, we collected three independent samples of venous blood, one of which was used for immediate routine blood work determination [total cholesterol (TC), LDL-C, HDL-C, triglyceride (TG), alanine aspartate transferase (AST), alanine aminotransferase (ALT), serum creatinine, ionogram]. The second and the third samples were centrifuged at 2000 rpm for 20 min and the resulting serum was stored according to the specifications provided by the assay manufacturers: for apoB and apoA, we stored the serum at −80 °C for a maximum of 2 months before measuring the apolipoproteins using a dedicated immunoturbidimetry standardized kit (Beijing Strong Biotechnologies, Inc., Beijing, China); for Lp(a) the serum was stored at −80 °C until we finished patient enrollment phase; then, all the measurements were performed at the same time, using an immunoturbidimetric assay (Randox Laboratories, Crumlin, UK) as well.

### 2.2. Statistical Analysis

We performed the statistical analysis using IBM SPSS Statistics version v26.0 software package (SPSS Inc., Chicago, IL, USA). A *p*-value < 0.05 was considered as the threshold for statistical significance throughout our analysis. For continuous variables, we calculated standard descriptive statistics (mean, standard deviation, minimum, maximum, and median values). Normal distribution of numerical data was tested using the Kolmogorov–Smirnov test. For normally distributed variables we used Student’s *t*-test to compare the difference in means between two groups and ANOVA test for multiple groups comparison. In case of statistically significant results, we further performed a post hoc Tukey correction, to adjust for multiple comparisons. For variables that were not normally distributed, we used the non-parametric Mann–Whitney test for two-group comparison and non-parametric Kruskal–Wallis test for multiple-group comparison. Also, because the Gensini score and Lp(a) did not present a normal distribution pattern, we log-transformed them, using the formulas ln(Gensini + 1) and ln(Lp(a)), before performing the statistical analysis. Categorical data were expressed as percentages and intergroup differences were tested using the Chi-square test. To facilitate interpretation of regression-based analyses, the apoB/apoA ratios were standardized (zApoB/ApoA) before statistical modeling. The standardization was performed using Z-score transformation and each individual value was centered around the cohort mean.

To evaluate the association between two continuous variables, we used Spearman’s correlation coefficient. Based on the reported coefficient (r), we were able to characterize the direction and the strength of the correlation. To further investigate the relationships between two correlated variables we constructed linear regression models. To test whether the studied biomarkers independently predicted CAD, we used logistic regression, and the results were reported as odds ratios (OR) with 95% confidence intervals. In order to determine whether the association between apoB/apoA ratio and the CAD was independent of other established CV risk factors, we also performed a multivariate logistic regression analysis. Therefore, we created two multivariate models: Model 1, which include zApoB/ApoA ratio, age, sex, BMI, DM, AF, and smoking status and Model 2, in which we included Lp(a), additionally to the previous model’s variables. Finally, the diagnostic performance of the apoB/apoA ratio was determined using the receiver operating characteristic (ROC) curve analysis, from which we reported the area under the curve (AUC) and also the cut-off values.

### 2.3. Ethics

Our study was conducted in accordance with the ethical principles stated in the Declaration of Helsinki, revised in 2013. A standard informed consent was signed by every patient, before enrollment and all data was processed anonymously. The research was approved by Ethics Committees of both University of Medicine and Pharmacy “Grigore T. Popa” Iași (no. 376/7.01.2024) and of the St. Spiridon Emergency Clinical Hospital (no. 82/25.09.2023).

## 3. Results

### 3.1. Baseline Characteristics

As seen in Table 1, patients in the S-CAD group were older than those without significant (62.94 ± 9.28 vs. 66 ± 9.33, *p* = 0.076) and predominantly male (59.4% vs. 69.2%, *p* = 0.267), even if the statistical significance was not reached. Apart from DM, no notable difference in terms of classical CV risk factors was observed between the two cohorts. Interestingly, patients with S-CAD had a considerable higher incidence of aortic atherosclerosis (25.8% vs. 74.2%, *p* = 0.000).

In terms of lipid profile, except for HDL-C and TG, all classical lipoproteins and Lp(a) showed important and statistically significant differences between the two groups. Contrary to HDL-C, the difference between means of apoA in the two studied groups was statistically significant (138.43 ± 27.05 vs. 128.90 ± 25.13, *p* = 0.048). By comparison, apoB was higher in the S-CAD (73 ± 23.47 vs. 93.01 ± 26.51, *p* = 0.000), which also resulted in a higher apoB/apoA ratio (0.53 ± 0.16 vs. 0.73 ± 0.18, *p* = 0.000).

### 3.2. ApoB/ApoA Ratio-Based Analysis

Further, we divided the enrolled patients into three groups based on apoB/apoA tertiles. A comparative analysis regarding clinical and paraclinical data across the three groups can be seen in Table 2. Interestingly, Lp(a), residual apoB (R-apoB), and LDL/apoB ratio did not show statistically significant differences between the three groups. However, Gensini score and log-Gensini score markedly increased in accordance with apoB/apoA tertiles (8.55 ± 19.6 vs. 14.57 ± 21.65 vs. 29.80 ± 27.78, *p* = 0.000). Even though statistical significance was not reached, male gender, DM, aortic atherosclerosis, and active smoking were more prevalent among patients with a higher apoB/apoA ratio.

**Figure 1:**
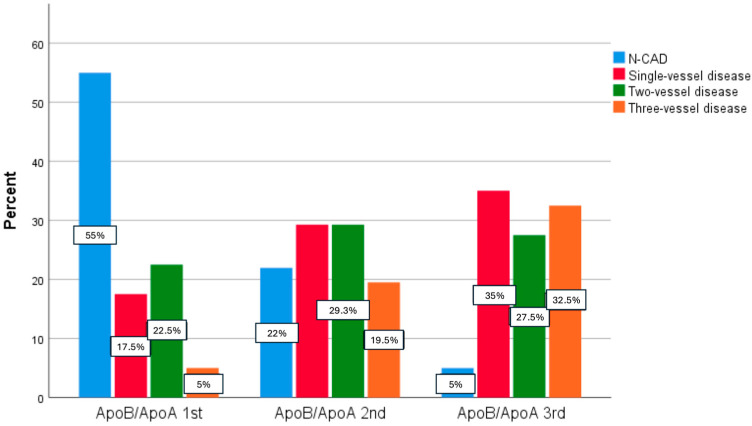
Distribution of CAD extent across apoB/apoA tertiles.

Furthermore, as the apoB/apoA ratio increased, so did the proportion of patients with three-vessel disease (5% vs. 19.5% vs. 32.5%, *p* = 0.000) and left man disease (5% vs. 7.3% vs. 20%, *p* = 0.031). Patients with no coronary artery disease (N-CAD) were more prevalent in the 2nd and 1st tertile groups, when compared with the 3rd tertile (55% vs. 22% vs. 5%, *p* = 0.000) (Table 2 and Figure 1).

### 3.3. Association Between apoB/apoA Ratio and the Severity of CAD

**Figure 2:**
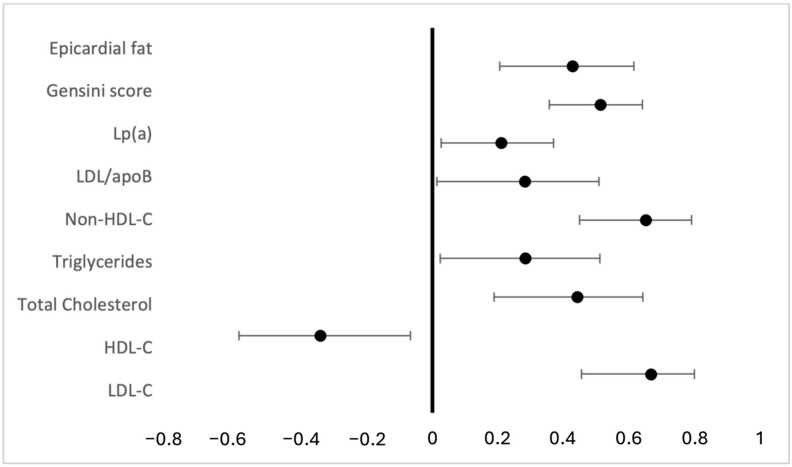
Forest plot of Spearman correlation coefficients between apoB/apoA ratio and different CV risk factors and Gensini score.

Spearmen’s correlation coefficient further supported the association between the apoB/apoA ratio and the severity of CAD, as it significantly correlated with the Gensini score (r = 0.513, *p* = <0.001, 95% CI: 0.357–0.641). Also, the apoB/apoA ratio positively correlated with classical pro-atherogenic lipid biomarkers and Lp(a), and it was inversely correlated with HDL-C (r = −0.344, *p* = 0.012, 95% CI: −0.591–−0.067). Furthermore, there was a moderate positive correlation between the apoB/apoA ratio and the presence of epicardial fat (r = 0.428, *p* = 0.001, 95% CI: 0.206–0.615), but no significant correlation with aortic atherosclerosis (Figure 2 and Supplementary Materials Table S1).

In linear regression analysis (Table 3), for each SD increase in the apoB/apoA ratio there was a 0.795 increase in the Gensini score. This model explained 26.6% of the changes in the Gensini score, showing a moderate, and statistically significant association between the two variables (*p* = 0.000, 95% CI: 0.559–1.030).

### 3.4. ApoB/ApoA Ratio as a Predictor for CAD

In the logistic regression analysis (Table 4), the apoB/apoA ratio showed a significant predictive power for both S-CAD presence and its severity. For each SD increase in the apoB/apoA ratio we observed a 2.509-fold increase in the odds for S-CAD (95% CI: 1.441–4.369, *p* = 0.001), 2.339-fold increase for three-vessel disease (95% CI: 1.427–3.892, *p* = 0.001), and 2.771-fold increase for left main disease (95% CI: 1.489–5.156, *p* = 0.001). Subsequently, we created a multivariate logistic regression model (Table 5, Model 1), which included several classical CV risk factors (age, sex, BMI, DM, AF, and smoking status), together with the apoB/apoA ratio. The resulting model showed a superior explanatory power for the presence of S-CAD (Nagelkerke R^2^ 0.443 vs. 0.186), three-vessel disease (Nagelkerke R^2^ 0.287 vs. 0.162), and left main disease (Nagelkerke R^2^ 0.351 vs. 0.189), when compared with the apoB/apoA ratio alone. Even after adjusting for these CV risk factors, the ratio remained statistically significant and predictive for all the dependent variables. Including Lp(a) in the first model (Table 5, Model 2) resulted only in a slight improvement in the model’s explanatory power (S-CAD: Nagelkerke R^2^ 0.443 vs. 0.452, left main disease: Nagelkerke R^2^ 0.351 vs. 0.353, three-vessel disease: Nagelkerke R^2^ 0.287 vs. 0.307).

As shown in Table 1, LDL-C was significantly higher in the S-CAD group and also showed a high correlation with the apoB/apoA ratio (Supplementary Materials Table S1). To reduce the confounding effect of LDL-C in CAD diagnosis, we further divided the enrolled patients according to LDL-C tertiles and performed the logistic regression analysis for apoB/apoA only in the lowest tertile group. As shown in Table 6, apoB/apoA ratio remained significantly associated with both S-CAD (OR 5.475, 95% CI: 1.550–19.326, Nagelkerke R^2^ 0.300, *p* = 0.008) and three-vessel disease (OR 15.871, 95% CI: 2.194–114.805, Nagelkerke R^2^ 0.481, *p* = 0.006).

**Figure 3:**
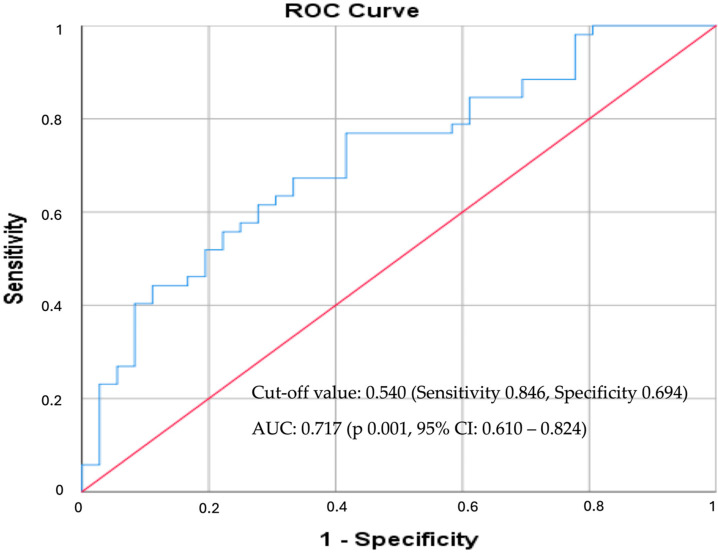
ROC curve expressing the association between apoB/apoA ratio and S-CAD. The red line represents the reference line. The blue line represents the ROC curve of the investigated biomarker (apoB/apoA ratio).

In the ROC curve analysis (Figure 3), the apoB/apoA ratio showed a strong discriminatory ability between patients with NS-CAD and S-CAD. With an AUC of 0.717 (95% CI: 0.610–0.824, *p* = 0.001), the ratio demonstrated a good diagnostic performance for S-CAD. The optimal cut-off value was 0.540, which provided a sensitivity of 84.6% and a specificity of 69.4%.

## 4. Discussion

This study explored the role of the apoB/apoA ratio as a biomarker for CAD in a cohort of 121 statin-treated patients with stable angina pectoris who underwent elective invasive coronary angiography. Furthermore, we tested whether Lp(a) provided any additional diagnostic value, over classic CV risk factors. Our results indicate that a higher apoB/apoA ratio may serve as an additional risk factor for CAD, as it was associated with both significant coronary stenosis and CAD severity.

Concerning the general characteristics, the demographic and clinical profile of our cohort was similar to other CV primary prevention studies [35]. Even if statistical significance was not reached, patients with S-CAD were older, predominantly men, and presented a higher prevalence of DM, stroke, and COPD. Interestingly, aortic atherosclerosis, described using transthoracic echocardiography, was significantly more prevalent in the S-CAD group. This finding supports the concept that atherosclerosis is a systemic process, affecting multiple arterial segments simultaneously. In the MESA cohort, abdominal aorta calcifications were predictive for both coronary artery calcifications and signs of peripheral artery disease, including increased intima–media thickness and decreased ankle-brachial index [36]. Furthermore, Han et al. proved on an impressive cohort of over 30,000 asymptomatic patients that thoracic aortic calcification may improve CV risk estimation and even help reclassify patients according to their CV risk [37].

In our analysis, patients with S-CAD presented significantly higher LDL-C and TC, which is not surprising, considering their already established association with CAD and CV risk. We could not find a statistically significant difference in HDL-C levels between the two groups; however, apoA was substantially lower among S-CAD patients. These two biomarkers are known to be highly correlated, and the superiority of apoA in predicting the CV risk has not been proven yet [38]. However, Florvall et al. demonstrated in their study that, at least in elderly man, apoA is a stronger marker for CV mortality than HDL-C and LDL-C. According to their research, in older patients, HDL’s quantity and quality may become divergent, so that HDL-C, which measures the total mass of cholesterol transported by this lipoprotein, may not reflect its functionality [39]. Still, the lack of statistical significance for HDL-C should be interpreted with caution, considering the relatively small number of patients in our cohort. Furthermore, apoB was considerably higher in the S-CAD. This finding aligns with the growing evidence that supports the role of apoB in CV risk assessment [40].

In the apoB/apoA ratio-based analysis, we observed that the Gensini score increased in accordance with the ratio’s tertiles. This linear correlation between apoB/apoA and the severity of CAD was further confirmed by the linear regression analysis. Our results align with previously published data. Hua et al. observed a similar trend, showing that CAD complexity increased stepwise with higher apoB/apoA ratio tertiles. However, in this analysis, the correlation with the Gensini score was observed only among patients with S-CAD. Also, patients with a history of acute coronary syndrome or prior coronary revascularization were not excluded from this analysis, so that the studied population does not qualify for a primary prevention cohort [41]. Liting et al. also found a significant correlation between the apoB/apoA ratio and Gensini score. In their analysis, apoB, apoB/apoA ratio, and non-HDL outperformed classic lipid biomarkers in predicting the severity of CAD and also in-hospital and long-term CV events [42]. Our paper does not provide a comparative analysis with other lipid biomarkers; however, in a previously published study from the same cohort, we showed that apoB outperformed LDL-C, non-HDL-C, and the LDL/apoB ratio. Although apoB demonstrated a good predictive ability for both the presence and the severity of CAD, its performance was lower, compared to apoB/apoA ratio [33].

Anatomical high-risk features, such as three-vessel disease or left main disease are known to be predictors of adverse CV events, irrespective of plaque hemodynamical significance. In a retrospective study, which included over 37,000 patients, the 1-year risk of myocardial infarction (MI) and death increased progressively with the number of diseased vessels [43]. Also, Bittencourt et al. showed that patients with extensive non-obstructive CAD had similar rates of MI and CV death, when compared with patients with S-CAD [44]. In the CONFIRM trial, non-obstructive left main disease was associated with an increased risk of CV events in women [45]. In our analysis, the apoB/apoA ratio was predictive for both left main and three-vessel disease, even in the multivariate analysis, after adjusting for other CV risk factors. In the Swedish trial AMORIS, the ratio was strongly associated with a higher risk of major adverse CV events (MACE) in both men and women. Furthermore, the age at which patients experienced MACE decreased progressively across apoB/apoA quintiles [21]. Similarly, in the INTERHEART trial, for each SD increase in the apoB/apoA ratio, the odds of developing MI increase 1.59 folds. These results outperformed those attributed to other classical lipid biomarkers [20]. In a more recent study, Zhang et al. observed that the apoB/apoA ratio was associated with an increased risk of MACE and one year readmission after percutaneous coronary revascularization. This association was particularly relevant among male participants, those with multivessel disease, and with LDL-C < 2.6 mmol/L [46]. In none of these studies the severity of CAD was estimated using a standardized score, like Gensini or SYNTAX.

Similarly to previous studies [41,46], in our analysis, standard lipid biomarkers (TC, TG, LDL-C, and non-HDL-C) increased progressively across the apoB/apoA tertiles. Also, TC, TG, LDL-C, and non-HDL-C showed a significant positive correlation with the ratio, whereas HDL-C was negatively correlated with it. Several published studies suggested that apoB or the apoB/apoA ratio does not provide substantial additional prognostic information, compared with standard lipid biomarkers. For example, in the UK Biobank analysis, the inclusion of TC and HDL-C in the multivariate analysis was enough to capture most of the lipid-associated CV risk [38]. In a similar way, Meisinger et al. showed that apoB/apoA and TC/non-HDL-C had similar predictive power for MI and cardiac death [47]. In another large Western European cohort, apoB and non-HDL-C performed comparably when it comes to CV events prediction, over a mean period of 11.4 years [48]. In our study, after restricting the analysis to the participants in the lowest LDL-C tertile, the apoB/apoA ratio remained substantially predictive for S-CAD and three-vessel disease, confirming that its diagnostic ability extends beyond classic lipid biomarkers. Larger prospective studies, like AMORIS or INTERHEART, already proved that this ratio can predict the CV event independent to standard lipid count; however, they did not include coronary angiography data, as they only tested for long term outcomes.

We observed a significant increase in Lp(a) levels in patients with S-CAD, compared to the N-CAD group. Also, Lp(a) increased progressively across apoB/apoA tertiles. These findings align with extensive epidemiological data and Mendelian randomization studies, that identified Lp(a) as an independent and causal risk factor for atherosclerotic disease [49,50]. Notably, we observed a moderate positive correlation between Lp(a) and the apoB/apoA ratio. This was expected, considering that each Lp(a) particle contains a covalently bound apoB and consequently this lipoprotein contributes directly to the pool of circulating apoB [51]. However, in multivariate analysis, the addition of Lp(a) to a model which included several traditional CV risk factors and the apoB/apoA ratio, resulted only in a marginal improvement in the predictive power. Most likely, this reflects that the apoB/apoA ratio already captures most of the atherogenic information provided by Lp(a). Our results do not contradict previous data, supporting Lp(a) as an independent risk factor for CV events. Beyond the pro-atherosclerotic properties mediated by its LDL component, Lp(a) also manifests pro-inflammatory and pro-thrombotic effects, which are attributed to apo(a) [52]. Given that our study evaluated a primary prevention cohort, without a history of ACS or coronary revascularization, the limited incremental contribution of Lp(a) to the multivariate models is explainable and likely reflects the lower overall event burden and the predominantly subclinical stage of disease in this population.

In our study, the apoB/apoA ratio was significantly associated with both the presence and the severity of CAD. Beyond its ability to discriminate between NS-CAD and S-CAD, a higher apoB/apoA ratio was linked to greater atherosclerotic burden, reflected by a higher prevalence of multivessel and left main disease. In one of our previously published papers, based on the same cohort, apoB proved to be superior to traditional lipid biomarkers, whereas the present analysis suggests that the apoB/apoA ratio provides additional discriminatory information beyond apoB alone. Consequently, this may be a strong argument supporting the lipid balance theory, stating that the CV risk does not only depend on the burden of atherogenic particles, but also on the balance between them and anti-atherogenic molecules [53]. Moreover, unlike LDL-C, which is routinely calculated based on the fasting lipid profile, apoB and apoA are measured directly using inexpensive and standardized methods [54]. This substantially reduces the influence of dietary intake and triglycerides on the measured concentrations, making apoB and, consequently, the apoB/apoA ratio, more reliable biomarkers, even in particular clinical contexts, such as metabolic syndrome or hypertriglyceridemia [4]. Lastly, the superiority of the apoB/apoA ratio may arise from a physiopathological perspective. Traditional lipid biomarkers like LDL-C and non-HDL-C measure the total cholesterol mass in atherogenic particles, which is known to be highly variable and to underestimate the CV risk in patients with cholesterol-depleted lipoproteins [55]. On the other hand, each circulating atherogenic lipoprotein contains a single molecule of apoB, making this apolipoprotein a more reliable indicator of the total atherogenic burden [7].

### Study Limitations

Our study has a series of limitations that we should acknowledge. First and foremost, the single-center design may affect generalizability. As we already know, the CV risk varies substantially across different regions, due to differences in genetics, lifestyle, socioeconomic factors, and healthcare access. However, our study is one of the first from Eastern Europe to analyze the association between the apoB/apoA ratio and CAD in an invasive coronary angiography-evaluated cohort. Secondly, the relatively limited sample size of our cohort restricts the diagnostic accuracy of the investigated biomarkers, as reflected by the ROC curve analysis. Consequently, the results should be interpreted as exploratory rather than definitive. Lastly, due to the observational and cross-sectional nature of the study, we could not determine the risk of future CV events inflicted by the studied biomarkers, which prevented us from evaluating their prognostic value.

Another limitation is that we did not specifically address the inter-observer variability, when it comes to stenosis evaluation. We used QCA assessment, which reduces substantially the subjectivity of the angiographic evaluation. However, a certain degree of intra-observer and inter-observant variability is still present, as this process still requires frame selection, lesion border definition, and manual contour detection.

## 5. Conclusions

Our proof-of-concept study demonstrated that the apoB/apoA ratio was associated with both the presence and the severity of CAD, independent to LDL-C. In this statin-treated cohort undergoing invasive coronary angiography, higher apoB/apoA values were also linked to high-risk coronary features, such as three-vessel disease and left main disease, further supporting its ability to reflect the balance between atherogenic and anti-atherogenic lipoproteins. In contrast, Lp(a) did not differ significantly between the studied groups, and it failed to bring any notable diagnostic power over the apoB/apoA ratio in the multivariate analysis.

Given the relatively small cohort size and the single-center design of this study, larger prospective cohorts are needed to validate an apoB/apoA ratio-based management of CAD. However, our results recommend this ratio as a solid potential biomarker for CAD diagnosis.

## Figures and Tables

**Table 1:** Demographic and biochemical findings of the studied cohort.

Parameter	Overall (n = 121)	NS-CAD (n = 69)	S-CAD (n = 52)	*p*-Value
Age (years)	64.2 ± 9.3	62.94 ± 9.28	66 ± 9.33	0.076
Male gender (%)	63.6%	59.4%	69.2%	0.267
Smoking (%)	56.2%	56.6%	55.8%	0.934
BMI (kg/m^2^)	30.17 ± 5.70	30.74 ± 5.93	29.42 ± 5.33	0.202
AF (%)	28.9%	27.53%	26.92%	0.956
DM (%)	24.8%	20.3%	30.8%	0.186
CKD (%)	21%	20.28%	21.15%	0.821
COPD (%)	9.1%	5.79%	13.46	0.147
Stroke/TIA (%)	9.9%	8.69	11.53%	0.605
Aortic atherosclerosis (%)	25.6%	25.8%	74.2%	**0.000**
Glycemia (mg/dL)	110.15 ± 29.83	109.7 ± 29.73	110.7 ± 30.25	0.862
AST (U/L)	24.45 ± 10.79	25.41 ± 13.42	23.40 ± 6.91	0.351
ALT (U/L)	24.74 ± 12.97	25.34 ± 14.77	24.06 ± 10.68	0.617
GGT (U/L)	49.07 ± 57.57	57.50 ± 69.41	40.16 ± 40.73	0.198
Creatinine (mg/dL)	0.88 ± 0.29	0.88 ± 0.28	0.87 ± 0.29	0.766
eGFR (mL/min/1.73 m^2^)	86.43 ± 18.35	86.12 ± 19.48	86.86 ± 16.80	0.837
Gensini Score	17.6 ± 24.73	2.63 ± 3.74	37.5 ± 26.72	**0.000**
Lipid parameters				
TC (mg/dL)	165.81 ± 41.97	155.60 ± 33.22	178.86 ± 48.29	**0.003**
LDL-C (mg/dL)	102.65 ± 38.38	92.76 ± 30.67	115.53 ± 43.63	**0.002**
HDL-C (mg/dL)	46.82 ± 12.97	47.19 ± 12.22	46.36 ± 13.97	0.737
TG (mg/dL)	107.48 ± 50.23	103.95 ± 46.76	112.08 ± 54.57	0.396
ApoB (mg/dL)	81.6 ± 26.65	73 ± 23.47	93.01 ± 26.51	**0.000**
ApoA (mg/dL)	134.33 ± 26.56	138.43 ± 27.05	128.90 ± 25.13	**0.048**
ApoB/ApoA	0.61 ± 0.19	0.53 ± 0.16	0.73 ± 0.18	**0.000**
Lp(a) (mg/dL)	17.19 ± 22.96	13.03 ± 21.70	22.64 ± 23.64	**0.022**

BMI: body-mass index; AF: atrial fibrillation; DM: diabetes mellitus; CKD: chronic kidney disease; COPD: chronic obstructive pulmonary disease; TIA: transient ischemic stroke; TC: total cholesterol; TG: triglycerides; eGFR: estimated glomerular filtration rate; LDL-C: low density lipoprotein cholesterol; HDL-C: high-density lipoprotein cholesterol; ApoB: apolipoprotein B; ApoA: apolipoprotein A; Lp(a): lipoprotein(a); Statistically significant *p*-values are highlighted in bold.

**Table 2:** Comparison of different clinical and paraclinical data between different apoB/apoA ratio tertiles.

Variable	ApoB/ApoA 1st (0.22–0.50)	ApoB/ApoA 2nd (0.50–0.68)	ApoB/ApoA 3rd (0.68–1.11)	*p*-Value
General characteristics				
Age	65.00 ± 9.02	64.39 ± 8.92	63.38 ± 10.35	0.740
Male gender (%)	60%	60.97%	70%	0.590
Smoking (%)	57.5%	46.34%	65%	0.234
BMI (kg/m^2^)	29.53 ± 4.27	30.54 ± 6.31	30.45 ± 6.36	0.681
DM(%)	22.5%	19.51%	32.5%	0.368
AF (%)	17.5%	31.7%	32.5%	0.206
CKD (%)	15%	26.82%	20%	0.426
Aortic atherosclerosis (%)	20%	24.39%	32.5%	0.430
Lipid biomarkers				
LDL-C	74.11 ± 18.53	100.82 ± 19.62	132.29 ± 44.95	**0.000**
HDL-C	51.58 ± 12.98	44.84 ± 9.46	44.36 ± 14.90	**0.027**
TC	141.84 ± 24.62	159.89 ± 26.63	194.31 ± 50.44	**0.000**
TG	90.65 ± 39.06	100.73 ± 37.71	129.85 ± 61.73	**0.001**
Non-HDLc	84.78 ± 30.38	106.63 ± 36.32	146.20 ± 49.51	**0.000**
Lp(a)	10.60 ± 13.40	18.93 ± 22.76	22.06 ± 28.98	0.069
log-Lp(a)	2.02 ± 0.90	2.44 ± 1.06	2.49 ± 1.17	0.064
ApoB	58.53 ± 15.73	82.44 ± 12.71	103.83 ± 26.81	**0.000**
R-ApoB	0.324 ± 0.475	0.500 ± 0.507	0.579 ± 0.500	0.079
ApoA	142.05 ± 27.08	137.85 ± 21.80	123.03 ± 27.32	**0.003**
LDL/ApoB	1.31 ± 0.44	1.23 ± 0.24	1.28 ± 0.26	0.598
Uric acid	5.62 ± 1.79	6.58 ± 1.96	5.76 ± 1.40	0.201
Creatinine	0.924 ± 0.357	0.860 ± 0.185	0.858 ± 0.300	0.531
eGFR	85.91 ± 19.79	84.65 ± 17.29	88.80 ± 18.24	0.615
Coronary artery disease				
Gensini	8.55 ± 19.60	14.57 ± 21.65	29.80 ± 27.78	**0.000**
log-Gensini	1.04 ± 1.40	1.91 ± 1.35	2.84 ± 1.28	**0.000**
N-CAD	22 (55%)	9 (22%)	2 (5%)	**0.000**
Single-vessel disease	7 (17.5%)	12 (29.3%)	14 (35%)	**0.000**
Two-vessel disease	9 (22.5%)	12 (29.3%)	11 (27.5%)	
Three-vessel disease	2 (5%)	8 (19.5%)	13 (32.5%)	
Left main disease	2 (5%)	3 (7.3%)	8 (20%)	**0.031**

Statistically significant *p*-values are highlighted in bold.

**Table 3:** Linear regression analysis between zApoB/ApoA ratio and log-Gensini score.

Biomarker	B	Std. Error	Standardized β	Adjusted R^2^	*p*-Value (95% CI)
apoB/apoA	0.795	0.119	0.522	0.266	**0.000** **(0.559–1.030)**
Constant	1.932	0.119			**0.000** **(1.697–2.166)**

Statistically significant *p*-values are highlighted in bold.

**Table 4:** Bivariate logistic regression for zApoB/ApoA ratio as predictor for S-CAD, three-vessel disease, and left main disease.

Biomarker	OR (95% CI)	Nagelkerke R^2^	*p*-Value
S-CAD	2.509 (1.441–4.369)	0.186	**0.001**
Three-vessel disease	2.339 (1.427–3.892)	0.162	**0.001**
Left main disease	2.771 (1.489–5.156)	0.189	**0.001**

Statistically significant *p*-values are highlighted in bold.

**Table 5:** Multivariate logistic regression including S-CAD, left main disease, and three-vessel disease as dependent variable. Reported OR for zApoB/ApoA ratio.

	OR (95% CI)	*p*-Value	Nagelkerke R^2^
S-CAD			
Model 1	4.476 (2.498–9.022)	<**0.001**	0.443
Model 2	4.149 (2.296–7.496)	<**0.001**	0.452
Left main disease			
Model 1	3.027 (1.404–6.526)	**0.005**	0.351
Model 2	2.949 (1.357–6.410)	**0.006**	0.353
Three-vessel disease			
Model 1	2.481 (1.416–4.347)	**0.001**	0.287
Model 2	2.297 (1.297–4.067)	**0.004**	0.307

Model 1: zApoB/ApoA ratio, age, sex, BMI, DM, AF, smoking status; Model 2: zApoB/ApoA ratio, age, sex, BMI, DM, AF, smoking status, Lp(a); statistically significant *p*-values are highlighted in bold.

**Table 6:** Bivariate logistic regression for zApoB/ApoA ratio as predictor for S-CAD and three-vessel disease for patients in the lowest LDL-C tertile (30–82 mg/dL).

Biomarker	OR (95% CI)	Nagelkerke R^2^	*p*-Value
S-CAD	5.474 (1.550–19.326)	0.300	**0.008**
Three-vessel disease	15.871 (2.194–114.805)	0.481	**0.006**

Statistically significant *p*-values are highlighted in bold.

## Data Availability

All data presented in this study are available within the article. The first author has all data used in this study.

## Supplementary Materials

The following supporting information can be downloaded at: https://www.mdpi.com/article/10.3390/medicina62020297/s1, Table S1. Spearman correlation between apoB/apoA ratio and different CV risk factor and Gensini score.
