# Supplementary material for: Diagnostic and Severity Assessment of Coronary Artery Disease Using ApoB/ApoA-I Ratio: Insights from a Statin-Treated Eastern European Cohort
Source: Medicina (Kaunas). 2026 Feb 2;62(2):297. doi: 10.3390/medicina62020297 (PMC12941951; doi:10.3390/medicina62020297)
Supplement: Supplementary file 1 [file medicina-62-00297-s001.zip › medicina-4081071-supplementary.pdf]

Table S1. Spearmen correlation between apoB/apoA ratio and different CV risk factor and Gensini score

| Variable | Spearman r | p-value (95% CI) |
|---|---|---|
| **LDL-C** | 0.667 | **<0.001** (0.455 - 0.800) |
| **HDL-C** | −0.344 | **0.012** (−0.591 - −0.067) |
| **TC** | 0.442 | **0.001** (0.188 - 0.643) |
| **TG** | 0.283 | **0.040** (0.024 - 0.512) |
| **Non-HDLc** | 0.652 | **<0.001** (0.449 - 0.792) |
| **LDL/apoB** | 0.282 | **0.041** (0.013 - 0.509) |
| **Lp(a)** | 0.210 | **0.022 (0.027 – 0.370)** |
| **Gensini Score** | 0.513 | **<0.001** (0.357 – 0.641) |
| **Uric Acid** | 0.022 | 0.877 (−0.244 - 0.309) |
| **Female sex** | −0.013 | 0.927 (−0.286 - 0.270) |
| **Age** | 0.018 | 0.899 (−0.277 - 0.293) |
| **Smoking** | 0.082 | 0.365 (-0.099 – 0.261) |
| **AF** | 0.116 | 0.207 (-0.066 – 0.278) |
| **Diabetes** | 0.220 | 0.114 (−0.063 - 0.470) |
| **Aortic Atherosclerosis** | 0.155 | 0.089 (-0.023 – 0.333) |
| **BMI** | 0.205 | 0.141 (−0.079 - 0.468) |
| **Epicardial Fat** | 0.428 | **0.001** (0.206 - 0.615) |

Statistically significant p-values are highlighted in bold.
